# Trace metal (Cd, Cu, Pb, Zn) fractionation in urban-industrial soils of Ust-Kamenogorsk (Oskemen), Kazakhstan—implications for the assessment of environmental quality

**DOI:** 10.1007/s10661-018-6733-0

**Published:** 2018-05-25

**Authors:** Michał Woszczyk, Waldemar Spychalski, Laura Boluspaeva

**Affiliations:** 1 Department of Quarternary Geology and Paleogeography, Adam Mickiewicz University, B. Krygowskiego 10, 61-680 Poznań, Poland; 2 Department of Soil Science, Poznań University of Life Sciences, Szydłowska 50, 60-656 Poznań, Poland; 3 Department of Ecology and Geography, Sarsen Amanzholov East Kazakhstan State University, Revolution street 2a, Ust-Kamenogorsk, Kazakhstan

**Keywords:** Trace metals, Soil contamination, Fractionation, Bioavailability, Kazakhstan

## Abstract

Ust-Kamenogorsk is one of the largest cities and industrial centers in Kazakhstan. Non-ferrous metallurgy (Zn–Pb smelter) has acted as a predominating industrial branch in the city since late 1940s. The industrial plants are situated directly adjacent to the residential area of the city which creates grievous ecotoxicological hazard. In the present paper, we aimed at assessing the trace metal pollution of top soils in Ust-Kamenogorsk and its potential threats to the local population. The top soils were sampled at 10 sites throughout the city center. We determined the physical and chemical properties of soils as well as the contents of Cd, Cu, Pb, and Zn. In addition, the soil samples were subjected to a five-step sequential extraction to ascertain the fractionation of trace metals. On this basis, we calculated the geoaccumulation index (I_geo_) and pollution load index (PLI) and assessed bioavailability of the elements. From our data, it emerged that the soils displayed a strong polymetallic pollution. PLI was as high as 33.4. Throughout the city, the trace metal contents exceeded the geochemical background and allowable values for residential, recreational, and institutional areas. The I_geo_ obtained were 3.7–6.5 for Cd, 1.5–4.7 for Cu, 2.8–5.7 for Pb, and 2.6–4.6 for Zn. The soils in Ust-Kamenogorsk displayed extremely high contamination with Cd, moderate to strong contamination with Pb and Zn, and low to moderate contamination with Cu. Cd and Pb were found to be the most bioavailable elements. The mobility of trace metals in the soils changed in the order Cd > Pb > Zn > Cu.

## Introduction

Soils of heavily urbanized and industrialized areas are prone to acute contamination with different biologically harmful substances among which trace metals (i.e., elements having concentrations below 100 mg kg^−1^ (IUPAC 2006), e.g., Cd, Cu, Hg, Ni, Pb, Zn) play a major role. These metals are primarily delivered to soils via atmospheric deposition of industrial dust originating from combustion of fossil fuel and gasoline as well as from non-ferrous metal smelting (Pacyna and Pacyna 2001).

On a global basis, combustion of gasoline releases 74% of anthropogenic Pb and non-ferrous metallurgy emits 70–73% of Cu, Cd, and Zn (Pacyna and Pacyna 2001). On a regional scale, however, the relative contributions from different sources in trace metal delivery can vary substantially. Appreciable amounts of Cd, Cu, Pb, and Zn to industrial and urban soils can be delivered by industrial sludge disposal, incineration of municipal waste, and car traffic (Ajmone-Marsan and Biasioli 2010). Martínez and Poleto (2014) showed that in large cities, vehicle traffic can be a major source of pollution with trace metals.

Accumulation of trace metals in top soils and their transformations to chemically active and mobile forms create considerable environmental hazard because it enables these elements to diffuse to ground waters and enter the food chain. Noxious effects of trace metals on soil biota and humans have been well documented (Hutton 1987; Kabata-Pendias 2011; Godt et al. 2006; Ettler 2015).

Notwithstanding the worldwide tendency to foster sustainable development and efficient cleaning systems for industrial waste and exhausts, the growing demand for metallurgical production in recent decades has resulted in enhanced emission of trace metals to the environment. Pacyna and Pacyna (2001) show that major portion of global anthropogenic emission of trace metals originates from developing countries in Asia. These countries account for 49% of global atmospheric emission of Cd, 50% Cu, 43% Pb, and 61% Zn, and four Asian countries (China, Japan, Korea, and Kazakhstan) are among the top ten emitters of Cd and Hg (Pacyna and Pacyna 2001; Li et al. 2009). Consequently, unlike in Europe and North America, the environmental pollution in Asia is constantly growing (Foell et al. 1995). Tian et al. (2015) showed that since the mid-twentieth century, the emission of Hg, As, Pb, Cd, Cu, and Zn and many other trace metals in China have displayed exponential increase. At the same time, Asian countries have had relatively low effective emission control and data on the distribution of pollutants in soils have been scarce, especially in central Asia.

In the current research, we aimed at depicting distribution and fractionation of four trace metals (Cd, Cu, Pb, and Zn) in the soils of Ust-Kamenogorsk, Kazakhstan. The city acts as a capital and the largest urban center (c.a. 300,000 inhabitants) of East Kazakhstan region (oblast). During the Soviet times, Ust-Kamenogorsk and its surroundings has become a major center of nonferrous mining and metallurgy (Pb, Zn, Ti, Mg, Be, Ta, Nb) as well as food processing and machine building industry. Since 2010 the mean annual production of Zn metal, refined Pb and Cu products in Ust-Kamenogorsk has been 302.5 MT yea^−1^, 107.9 MT year^−1^ and 58.2 MT year^−1^, respectively (http://www.kazzinc.com/en/Production). Industrial plants in the city are located adjacent to densely inhabited areas and therefore, the indigenous population is directly exposed to heavy-metal-laden industrial emissions. In 2009 the total atmospheric release of toxic pollutants in the city (including Pb, Cu and Cd species) was 65.5 × 10^3^ tons (Boluspaeva et al. 2011).

Our approach was to assess the pollution of the city’s soils via calculating the geoaccumulation (I_geo_) and pollution load (PLI) indices as well as analysis of chemical fractionation of Cd, Cu, Pb, and Zn in relation to chemical and physical composition of soils (pH_soil_, grain size composition, Fe and Mn contents). The fractionation was analyzed using the procedure by Tessier et al. (1979), in which five fractions were identified: exchangeable, acid-extractable, reducible, oxidizable, and residual. Metals held in the exchangeable fraction are only loosely adsorbed onto sediment particles. The acid-extractable fraction includes carbonate-bound or carbonate co-precipitated metals and is highly sensitive to variations in soil pH. The metals partitioned to exchangeable and acid-soluble forms are regarded bioavailable (Zhou et al. 2017). Reducible fraction is composed of elements associated with colloidal hydrated iron and manganese oxides, which are very efficient scavengers for trace metals, and metalloids. In principle, the reducible fraction remains stable under oxidizing conditions (Joksič et al. 2005). The oxidizable fraction encompasses organic matter-bound (chelated complexes) and sulfide-associated metals and is best preserved under anoxic conditions. Metals held within crystalline matrices of primary and secondary aluminosilicates are referred to as residual form. The knowledge of distribution of trace metals and their potential bioavailability in the soils can act as a basis for assessment of ecotoxicological risk and a trigger for implementation of recovery plan.

### Study area

**Fig. 1:**
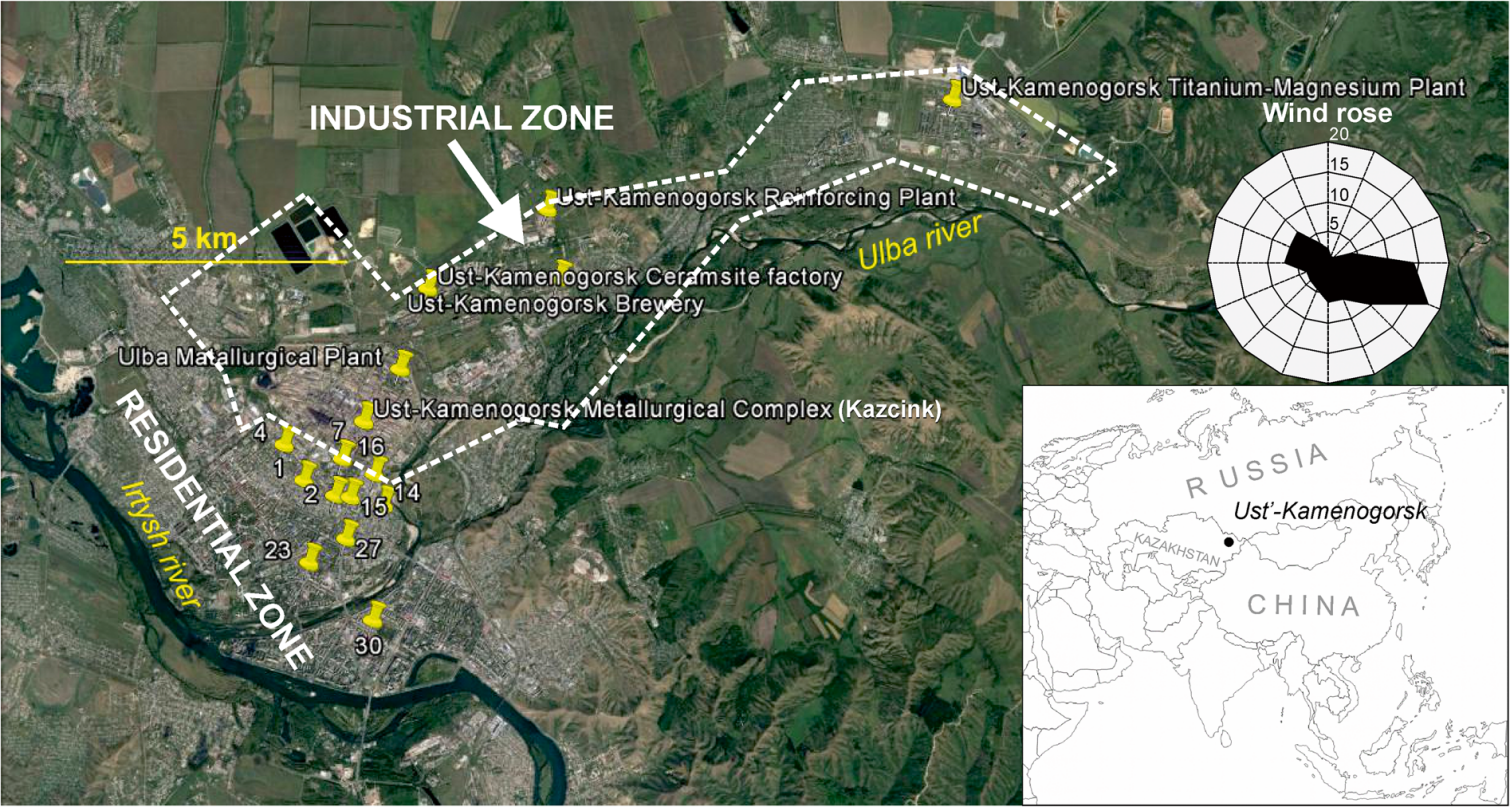
Location of the soil sampling sites (indicated by numbers) and major industrial plants in Ust-Kamenogorsk (indicated by names). Dashed line encircles an industrial zone of the city. Wind rose was drawn on the basis of meteorological data taken from https://www.meteoblue.com/en/weather/forecast/modelclimate/ust-kamenogorsk_kazakhstan_1520316

Ust-Kamenogorsk (Oskemen) is situated in the NE Kazakhstan in the foothills of the Altay and at a confluence of the Irtysh and the Ulba rivers. The climate of Ust-Kamenogorsk region is temperate continental. According to the Köppen-Geiger approach, the climate is classified as Dfb, i.e., as snow (D), fully humid (f) with warm summers (b) (Kottek et al. 2006). Mean monthly temperatures vary in a broad range from − 16 °C in February to + 21 °C in July while the average annual temperature is + 2 °C. Monthly precipitation ranges from 21 mm in August to 65 mm in November and the total annual sum is 466 mm. Prevailing winds (approximately 41% of windy days) are from E–SE direction (Fig. 1), and consequently a large part of the city lies a bit outside the major transport pathway of exhausts from the industrial plants. The soils of the study area are primarily represented by haplic and gleyic chernozems (Boluspaeva et al. 2011).

## Methods

### Soil sampling

The 1–1.5 kg soil samples were taken from 10 sites distributed throughout the major residential part of the city, as shown in Fig. 1. Each sample was a composite of 10–15 subsamples taken with a soil sampler from 0 to 20 cm layer after removal of the topmost herbaceous cover. Such depth interval is accepted worldwide for top soil (Escarré et al. 2011).

### Laboratory analyses

**Fig. 2:**
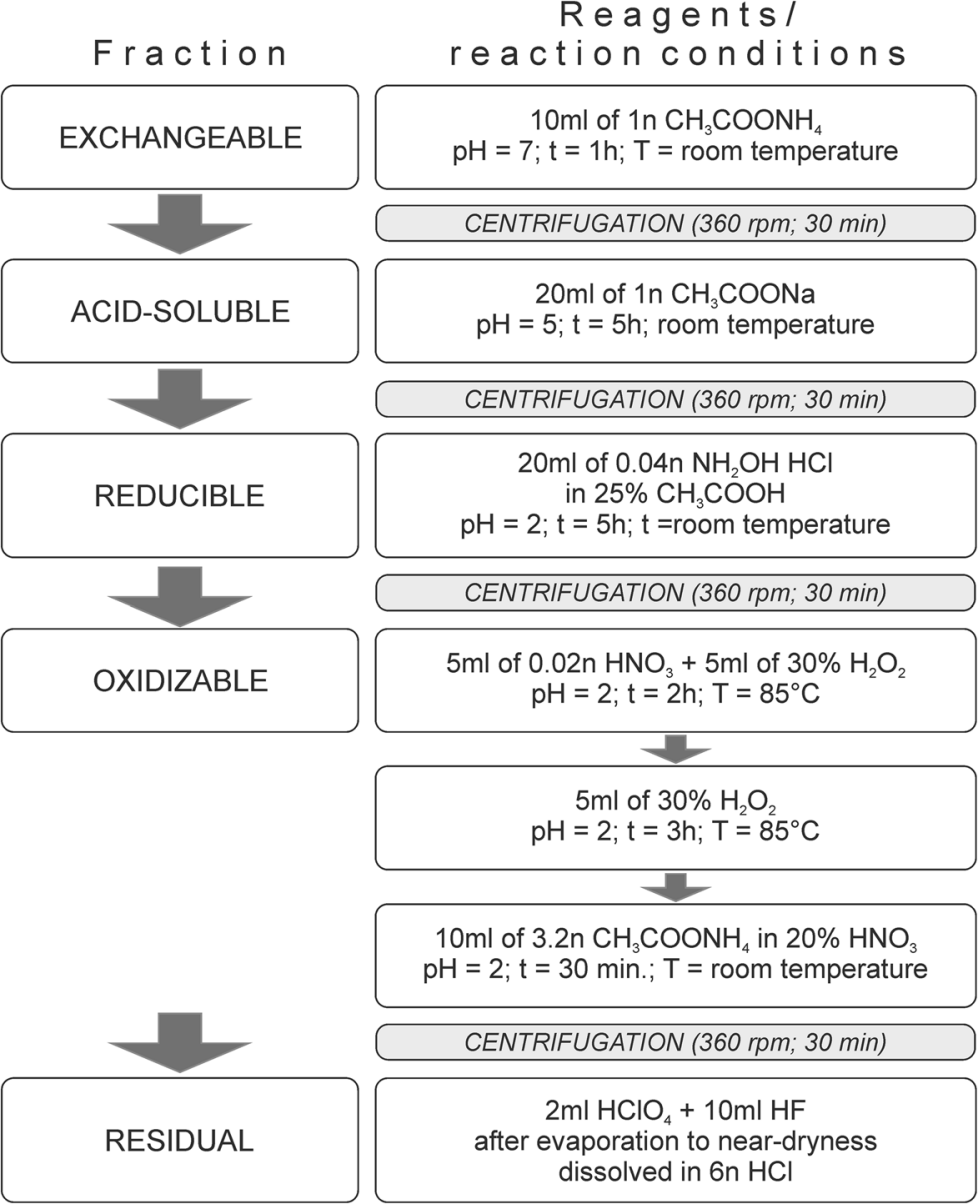
Procedure of sequential extraction used in the study

Prior to analyses, the samples were homogenized in an agate mortar and air dried at room temperature. Grain size composition was determined using a laser particle size analyzer Mastersizer 2000 with Hydro MU dispersion unit (Malvern) (Ryżak and Bieganowski 2011). Chemical composition of the soils was analyzed in < 2 mm fraction. The soil pH (pH_soil_) was measured potentiometrically in water extract and in 0.01 M CaCl_2_ solution with a soil/extractant ratio of 1:1 and 1:5, respectively. The total organic carbon (TOC) and total nitrogen (TN) were analyzed using a VarioMax elemental analyzer (Elmentar). For TOC analyses, the samples were decarbonated with 0.1 M HCl. For pseudototal contents (C_PTOT_) of trace metals, the homogenized samples were digested in aqua regia at room temperature for 12 h and then at 200 °C for 2 h. Sequential extraction of Cd, Cu, Zn, and Pb was performed as recommended by Tessier et al. (1979). This methodology allowed us to distinguish five element fractions: exchangeable, acid-soluble, reducible, oxidizable, and residual. The procedure of sequential extraction was depicted in Fig. 2. For centrifugation, we used Hermle Z513 centrifuge. The concentrations of the elements were measured using FAAS Varian SpectrAA 220FS.

### Analytical quality control

**Fig. 3:**
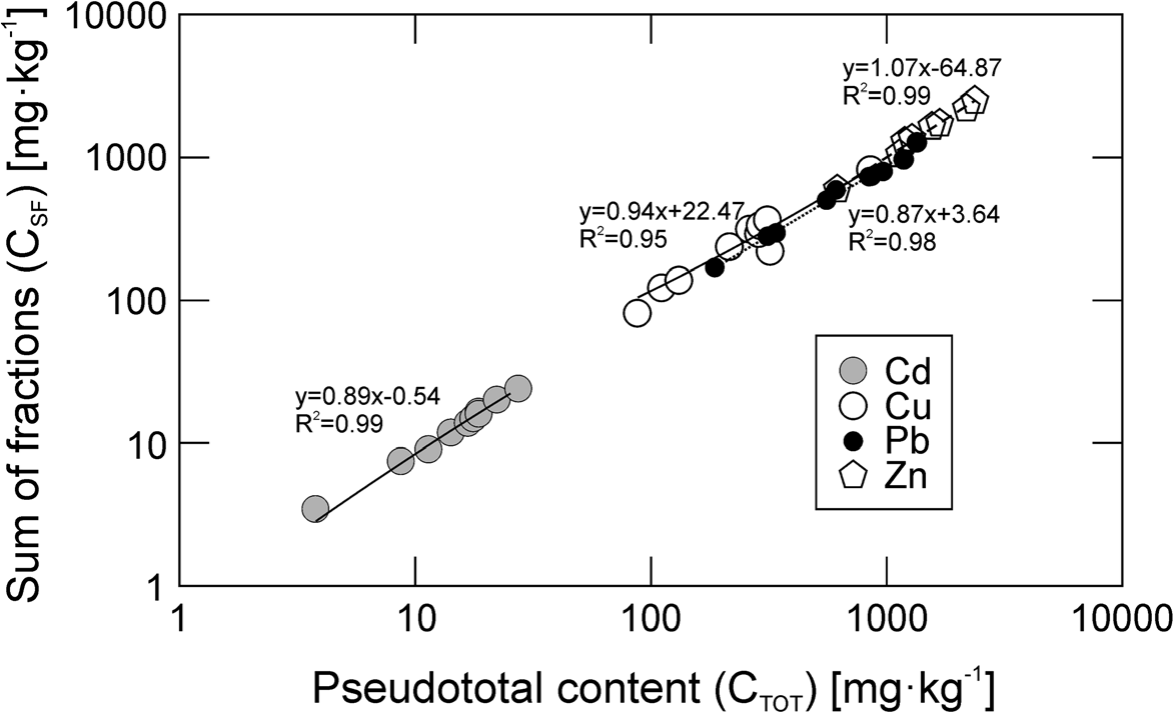
Relationship between pseudototal (aqua regia-extracted; C_PTOT_) contents and the summed contents of sequentially extracted fractions (C_SF_) of trace metals in the soils of Ust-Kamenogorsk. Due to large differences in the metal contents, the axes were log-transformed. C_PTOT_ and C_SF_ are strongly positively correlated. In majority of samples, C_PTOT_ were higher than C_SF_. In 28 out of 40 samples, the differences between C_SF_ and C_PTOT_ were ± 15%, while in 12 samples C_PTOT_-C_SF_ > 15%

Each sample was analyzed in duplicate. The quality of the measurements was controlled by using *Metals in sewage sludge SQC001S-50G* (RTC; USA) certified reference material. Recovery was between 94 and 98%. In majority of samples, C_PTOT_ was higher than the sum of fractions (C_SF_). In 28 out of 40 samples, the differences between C_SF_ and C_PTOT_ was ± 15%, while in 12 samples, C_PTOT_ − C_SF_ > 15%. The C_PTOT_ and C_SF_ values were strongly positively correlated with *R*^2^ between 0.95 and 0.99 (Fig. 3).

### SEM

Sample nos. 1 and 2 were examined using an S-3700N Hitachi scanning electron microscope (SEM) with EDS Noran SIX system for chemical microanalysis. The samples were operated at low vacuum conditions. Sample no. 1 was selected for SEM-EDS owing to the highest contamination of all studied samples. Sample no. 2 was selected owing to its contrasting fractionation of trace metals (the highest relative contribution of residual fraction).

### Assessment of soil pollution

To assess the trace metal pollution of soils, we used geoaccumulation index (I_geo_) and pollution load index (PLI). I_geo_ was calculated with the formula by (Müller 1969):

1 $$ {I}_{\mathrm{geo}}={\log}_2\left(\frac{C_n}{1.5\bullet {B}_n}\right) $$

where

*C*_*n*_: measured total concentration of the element in a sample
*B*_*n*_: local geochemical background.

The *B*_*n*_ values, taken from (Boluspaeva et al. 2011), were 0.2 mg kg^−1^ for Cd, 21.4 mg kg^−1^ for Cu, 17.8 mg kg^−1^ for Pb, and 67.4 mg kg^−1^ for Zn. The degree of contamination was assessed qualitatively based on the following criteria (Müller 1969):

1. I_geo_ < 0 uncontaminated soil
2. 0 < I_geo_ < 1 uncontaminated to moderately contaminated soil
3. 1 < I_geo_ < 2 moderately contaminated soil
4. 2 < I_geo_ < 3 moderately to strongly contaminated soil
5. 3 < I_geo_ < 4 strongly contaminated soil
6. 4 < I_geo_ < 5 strongly to extremely contaminated soil
7. I_geo_ > 5 extremely contaminated soil

Pollution load index (PLI) was derived from the equation given by Bhuiyan et al. (2010):

2 $$ PLI={\left({CF}_1\bullet {CF}_2\bullet {CF}_3\bullet \dots \bullet {CF}_n\right)}^{\frac{1}{n}} $$

where *CF* represents contamination factor for the metals analyzed

3 $$ CF=\frac{{\overline{C}}_n}{B_n} $$

and ${\overline{C}}_n$ is the average metal content for all sampling sites.

The Pearson correlation coefficients (*r*) and their corresponding probability values were calculated using Past 3.16 (Hammer et al. 2001).

## Results and discussion

### Physical and chemical properties of the soils studied

The soil matrix was predominantly sandy loam, except for site no. 1, where loamy silt was obtained (Table 1). The TOC content range from 1.6 to 5.6%, while TN varies between 0.1 and 0.5% (Table 1). The TOC/N is 9.8–12.8 (Table 1) providing evidence for high degree of humification of soil organic matter and its enhanced reactivity. Fe_tot_ and Mn_tot_ range from 17,463.5 to 26,961.2 mg kg^−1^ (1.7 and 2.7%, respectively) and from 439.7 to 521.6 mg kg^−1^ (0.04 and 0.05%, respectively) (Table 1). Fe is depleted and Mn is close to the mean continental crust values (3.49 and 0.05%, respectively; Taylor and McLenan 1995) which thus points to the natural origin of Fe and Mn in the soils. The soil pH (pH_soil_) in majority of samples is slightly alkaline, and in one site (site no. 2), slightly acidic conditions occurred (Table 1).

**Table 1:** Physical and chemical properties of soils studied

Sampling site	Grain size distribution						Chemical composition				
	Sand	Silt	Clay	Class	TOC	TN	Mn_tot_	Fe_tot_	TOC/N	pH_soil_	
	[%]				[%]		mg kg^−1^			H_2_O	CaCl_2_
1	38.73	56.50	4.77	Loamy silt	4.83	0.45	485.3	26,961.2	10.7	7.40	7.20
2	67.12	29.01	3.87	Sandy loam	4.81	0.43	439.7	21,223.4	11.2	6.32	6.22
4	63.68	32.38	3.94	Sandy loam	3.04	0.30	487.4	25,266.9	10.3	7.65	7.30
7	65.35	30.37	4.29	Sandy loam	5.55	0.50	505.1	26,576.0	11.2	7.80	7.54
14	66.36	29.07	4.57	Sandy loam	1.74	0.15	521.6	22,287.5	11.3	7.95	7.55
15	62.48	33.19	4.33	Sandy loam	1.65	0.13	499.3	18,932.5	12.8	7.44	7.11
16	79.86	17.71	2.43	Sandy loam	2.15	0.19	446.2	17,463.5	11.4	7.41	7.14
23	52.57	42.70	4.73	Sandy loam	1.60	0.14	481.6	25,845.7	11.2	7.50	7.19
27	70.52	26.54	2.94	Sandy loam	5.30	0.47	465.9	24,433.7	11.2	7.88	7.55
30	56.84	38.11	5.05	Sandy loam	2.39	0.24	515.2	26,950.2	9.8	7.35	7.25

### Trace metal contents and fractionation in the soils of Ust-Kamenogorsk

**Fig. 4:**
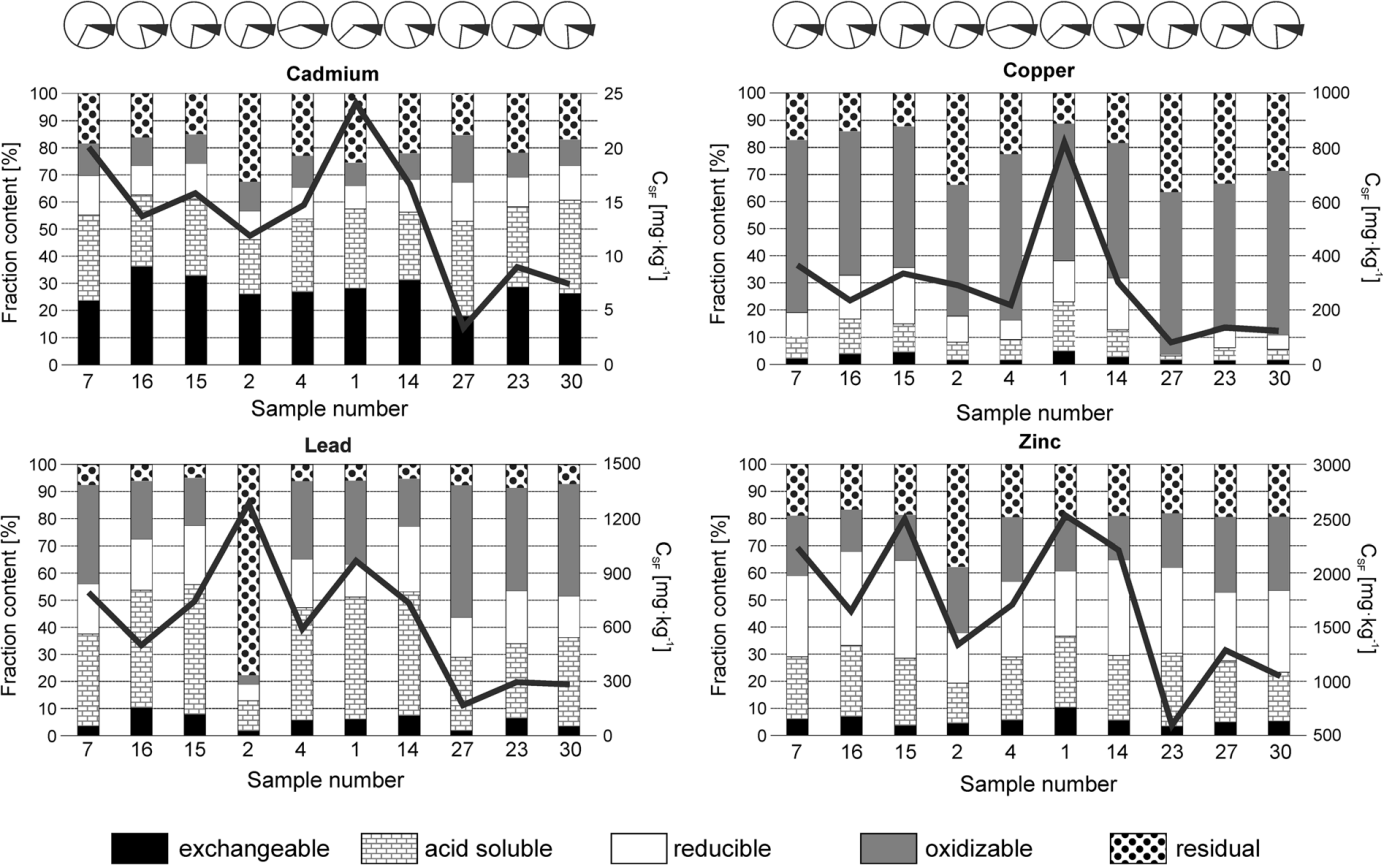
Relative distribution of sequentially extracted fractions of trace metals (stacked columns) and the contents of trace metals (sum of sequentially extracted fractions; C_SF_; solid lines) in the soil samples studied. Samples are ordered according to increasing distance from the major industrial plant (Kazcink) in Ust-Kamenogorsk. Circle diagrams above the upper panels indicate the position of the each site in relation to Kazcink (center of a circle) and predominating wind direction (black triangle)

The contents of trace metals (C_PTOT_) in the soils throughout a major part of the city of Ust-Kamenogorsk are very high and range from 3.8 and 27.4 mg kg^−1^ for Cd (Table 2), 87.9 and 856.3 mg kg^−1^ for Cu (Table 3), 187.2 and 1347.7 mg kg^−1^ for Pb (Table 4), and 625.3 and 2406.3.00 mg kg^−1^ for Zn (Table 5). The trace metal contents show positive intercorrelations with *r* values between 0.64 and 0.92 (Table 6) typical for local source of trace metals (Davies 1992). At the same time, the abundances of trace metals are weakly dependent on the physical and chemical properties of soils (such as Fe and Mn contents, pH_soil_, and granulometric fractions) (Table 6) and display an overall decrease with increasing distance from the major industrial complex in the city (Fig. 4). This observation agrees with Karczewska (1996) and Escarré et al. (2011) who concluded on the governing role of distance from the source and local wind directions for distribution of trace metals over industrialized zones. However, the trace metal distribution pattern is reasonably irregular (Fig. 4), and the correlation between trace metals and distance from the source of pollution in the soils of Ust-Kamenogorsk is relatively low, with *r* ranging from − 0.39 to − 0.64. We believe that the irregularities arise from spatial variability of TOC content and textural features of the soils. This would explain distinct enrichment in Cd, Cu, Pb, and Zn in site nos. 1 and 7 located a bit off the predominating winds. In these sites, the enhanced accumulation of trace metals might be supported by the highest silt and/or TOC content of all the sites studied (Table 1).

**Table 2:** The pseudototal contents, fractionation, and geoaccumulation index (I_geo_) of Cd in the samples analyzed

Sampling site	Distance from pollution source^a^	Cd content (±standard deviation)							I_geo_^b^
		Pseudototal	Exchangeable	Acid-soluble	Reducible	Oxidizable	Residual	Sum	
	[km]	[mg kg^−1^]							
1	1.48	27.4 ± 1.3	6.8 ± 0.1	7.0 ± 0.04	2.1 ± 0.1	2.0 ± 0.3	6.1 ± 0.5	24.0 ± 0.6	6.5
2	1.38	14.2 ± 0.7	3.1 ± 0.04	2.4 ± 0.1	1.3 ± 0.01	1.3 ± 0.1	3.9 ± 0.4	11.9 ± 0.4	5.6
4	1.46	17.6 ± 0.8	4.0 ± 0.1	3.9 ± 0.3	1.7 ± 0.2	1.7 ± 0.1	3.4 ± 0.7	14.7 ± 0.9	5.9
7	0.76	22.3 ± 1.4	4.7 ± 0.1	6.3 ± 0.2	2.9 ± 0.3	2.4 ± 0.2	3.7 ± 0.1	20.1 ± 0.5	6.2
14	1.51	18.6 ± 0.7	5.2 ± 0.1	4.2 ± 0.15	2.0 ± 0.1	1.6 ± 0.1	3.7 ± 0.3	16.6 ± 0.4	6.0
15	1.34	18.6 ± 1.3	5.2 ± 0.1	4.5 ± 0.3	2.1 ± 0.1	1.7 ± 0.1	2.4 ± 0.1	15.9 ± 0.4	6.0
16	0.94	16.8 ± 1.2	5.0 ± 0.2	3.6 ± 0.05	1.5 ± 0.2	1.4 ± 0.3	2.2 ± 0.7	13.7 ± 0.8	5.8
23	2.61	11.4 ± 0.6	2.6 ± 0.2	2.7 ± 0.1	1.0 ± 0.1	0.8 ± 0.1	2.0 ± 0.15	9.0 ± 0.3	5.2
27	2.06	3.8 ± 0.2	0.6 ± 0.01	2.2 ± 0.1	0.5 ± 0.1	0.6 ± 0.1	0.5 ± 0.05	3.5 ± 0.1	3.7
30	3.44	8.7 ± 0.6	2.0 ± 0.04	2.6 ± 0.1	1.0 ± 0.02	0.7 ± 0.1	1.3 ± 0.01	7.45 ± 0.2	4.9

^a^Measured from Ust-Kamenogorsk metallurgical complex (Kazcink); see Fig. 1

^b^Pseudototal content used as *C*_*n*_ in formula (1)

**Table 3:** The pseudototal contents, fractionation, and geoaccumulation index (I_geo_) of Cu in the samples analyzed

Sampling site	Distance from pollution source^a^	Cu content (± standard deviation)							I_geo_^b^
		Pseudototal (C_PTOT_)	Exchangeable	Acid-soluble	Reducible	Oxidizable	Residual	Sum (C_SF_)	
	[km]	[mg kg^−1^]							
1	1.48	856.3 ± 48.0	40.3 ± 9.8	147.8 ± 72.7	124.8 ± 35.9	414.0 ± 71.3	92.5 ± 2.3	819.5 ± 108.4	4.7
2	1.38	286.3 ± 13.1	4.5 ± 0.2	19.4 ± 0.5	27.4 ± 0.8	141.0 ± 7.4	97.9 ± 5.6	290.2 ± 9.4	3.2
4	1.46	321.3 ± 23.5	3.5 ± 0.2	16.7 ± 1.6	15.5 ± 0.8	133.7 ± 43.3	49.5 ± 3.1	218.9 ± 43.4	3.3
7	0.76	311.5 ± 17.8	8.7 ± 0.4	29.3 ± 0.8	32.0 ± 2.0	233.2 ± 6.5	62.7 ± 0.5	365.5 ± 6.9	3.3
14	1.51	262.3 ± 16.8	8.8 ± 0.01	31.1 ± 1.2	59.5 ± 5.3	155.7 ± 12.5	47.8 ± 0.2	312.9 ± 13.6	3.0
15	1.34	291.3 ± 13.7	15.4 ± 5.1	34.0 ± 6.6	69.8 ± 6.5	174.3 ± 2.0	40.6 ± 0.9	334.0 ± 10.8	3.2
16	0.94	215.4 ± 10.6	9.4 ± 0.3	29.7 ± 2.8	38.2 ± 3.0	125.0 ± 6.5	32.9 ± 6.1	235.2 ± 9.9	2.7
23	2.61	131.5 ± 8.2	2.0 ± 0.4	6.6 ± 0.6	9.3 ± 2.2	73.5 ± 1.0	45.9 ± 3.0	137.2 ± 4.0	2.0
27	2.06	87.9 ± 4.9	1.4 ± 0.3	1.2 ± 0.3	0.5 ± 0.6	48.2 ± 0.8	29.6 ± 0.6	80.9 ± 1.2	1.5
30	3.44	111.3 ± 7.0	2.0 ± 0.1	4.85 ± 0.7	6.5 ± 0.6	74.0 ± 8.2	35.0 ± 1.8	122.4 ± 8.5	1.8

^a^Measured from Ust-Kamenogorsk metallurgical complex (Kazcink); see Fig. 1

^b^Pseudototal content used as *C*_*n*_ in formula (1)

**Table 4:** The pseudototal contents, fractionation, and geoaccumulation index (I_geo_) of Pb in the samples analyzed

Sampling site	Distance from pollution source^a^	Pb content (±standard deviation)							I_geo_^b^
		Pseudototal (C_PTOT_)	Exchangeable	Acid-soluble	Reducible	Oxidizable	Residual	Sum (C_SF_)	
	[km]	[mg kg^−1^]							
1	1.48	1183.6 ± 105.3	59.8 ± 2.8	436.0 ± 28.1	116.9 ± 12.9	297.1 ± 5.5	55.8 ± 1.1	965.6 ± 31.5	5.5
2	1.38	1347.7 ± 95.7	26.0 ± 2.8	142.2 ± 4.7	76.8 ± 4.5	42.5 ± 0.2	992.3 ± 66.8	1279.7 ± 62.7	5.7
4	1.46	608.4 ± 38.9	34.0 ± 1.1	245.5 ± 6.8	106.1 ± 12.0	168.0 ± 18.2	36.3 ± 1.1	589.9 ± 22.9	4.5
7	0.76	967.2 ± 61.9	29.0 ± 1.7	269.1 ± 5.0	146.7 ± 6.1	287.8 ± 16.4	58.8 ± 1.8	791.4 ± 18.3	5.2
14	1.51	842.1 ± 45.5	55.7 ± 1.3	363.3 ± 32.7	146.4 ± 5.1	128.3 ± 9.1	38.0 ± 0.7	731.7 ± 34.4	5.0
15	1.34	868.7 ± 42.6	59.9 ± 5.5	354.2 ± 37.9	160.2 ± 14.1	131.1 ± 8.1	35.8 ± 2.5	741.2 ± 41.7	5.0
16	0.94	557.2 ± 31.2	53.1 ± 2.7	215.5 ± 41.4	93.6 ± 8.2	107.3 ± 5.3	29.5 ± 4.2	499.0 ± 42.9	4.4
23	2.61	340.1 ± 13.3	19.5 ± 2.4	80.8 ± 37.2	57.5 ± 1.8	111.8 ± 2.3	25.0 ± 1.4	294.6 ± 37.4	3.7
27	2.06	187.2 ± 8.6	3.3 ± 1.3	46.0 ± 3.4	25.2 ± 2.6	82.5 ± 0.00	12.8 ± 0.3	169.8 ± 4.4	2.8
30	3.44	308.0 ± 17.2	10.1 ± 0.4	91.2 ± 16.7	42.8 ± 4.5	115.2 ± 10.6	19.8 ± 1.1	279.1 ± 20.3	3.5

^a^Measured from Ust-Kamenogorsk metallurgical complex (Kazcink); see Fig. 1

^b^Pseudototal content used as *C*_*n*_ in formula (1)

**Table 5:** The pseudototal contents, fractionation, and geoaccumulation index (I_geo_) of Zn in the samples analyzed

Sampling site	Distance from pollution source^a^	Zn content (± standard deviation)							I_geo_^b^
		Pseudototal (C_PTOT_)	Exchangeable	Acid-soluble	Reducible	Oxidizable	Residual	Sum (C_SF_)	
	[km]	[mg kg^−1^]							
1	1.48	2406.3 ± 187.7	261.4 ± 9.0	661.7 ± 65.2	609.1 ± 24.8	465.1 ± 8.0	523.7 ± 15.6	2521.0 ± 72.5	4.6
2	1.38	1280.6 ± 87.1	62.0 ± 3.3	199.1 ± 0.3	252.5 ± 15.8	325.5 ± 17.8	509.9 ± 31.7	1349.0 ± 39.8	3.7
4	1.46	1689.3 ± 126.7	98.0 ± 4.2	397.7 ± 1.6	477.1 ± 63.4	401.7 ± 91.6	334.6 ± 34.2	1709.2 ± 116.6	4.1
7	0.76	2159.5 ± 153.3	135.6 ± 0.3	513.5 ± 1.8	673.7 ± 35.5	489.1 ± 26.9	424.7 ± 9.9	2236.7 ± 45.7	4.4
14	1.51	2198.2 ± 112.1	125.2 ± 2.3	528.6 ± 4.0	786.5 ± 47.0	351.7 ± 35.4	422.1 ± 6.0	2214.1 ± 59.4	4.4
15	1.34	2368.9 ± 116.1	97.3 ± 11.1	611.8 ± 88.4	906.1 ± 59.5	414.5 ± 27.8	466.1 ± 11.7	2495.7 ± 111.2	4.6
16	0.94	1568.8 ± 81.6	117.1 ± 1.0	433.4 ± 35.4	572.9 ± 79.9	251.5 ± 0.2	275.7 ± 51.3	1650.6 ± 101.3	4.0
23	2.61	1195.2 ± 86.1	43.8 ± 3.0	347.1 ± 3.1	408.7 ± 13.7	255.6 ± 9.9	232.0 ± 16.4	1287.1 ± 23.9	3.6
27	2.06	625.3 ± 48.8	29.7 ± 1.1	135.1 ± 0.8	151.5 ± 17.5	166.3 ± 2.5	117.1 ± 6.0	599.7 ± 18.8	2.6
30	3.44	1140.8 ± 92.4	55.2 ± 1.4	188.9 ± 60.1	314.8 ± 5.4	283.6 ± 13.5	201.2 ± 12.7	1043.7 ± 63.2	3.5

^a^Measured from Ust-Kamenogorsk metallurgical complex (Kazcink); see Fig. 1

^b^Pseudototal content used as *C*_*n*_ in formula (1)

**Table 6:** Pearson’s correlations coefficients (*r*) between trace metals and soil parameters. Probability values for *r* given in brackets. Statistically significant correlations marked in italics

	Cd_ptot_	Cu_ptot_	Pb_ptot_	Zn_ptot_	Mn_ptot_	Fe_ptot_	Sand	Silt	Clay	C_org_	pH_soil_ (_CaCl2)_
Cd_ptot_		*0.83* (*p* = 3·10^−3^)	*0.74* (*p* = 0.01)	*0.92* (*p* = 1·10^−3^)	0.21 (*p* = 0.57)	− 0.01 (*p* = 0.99)	− 0.36 (*p* = 0.29)	0.36 (p = 0.29)	0.27 (*p* = 0.42)	0.09 (*p* = 0.70)	0.00 (*p* = 0.86)
Cu_ptot_			*0.68* (*p* = 0.03)	*0.68* (*p* = 0.03)	0.03 (*p* = 0.93)	0.21 (p = 0.57)	− 0.64 (*p* = 0.07)	0.65 (*p* = 0.06)	0.28 (*p* = 0.39)	0.33 (*p* = 0.38)	− 0.09 (*p* = 0.80)
Pb_ptot_				*0.64* (*p* = 0.04)	− 0.12 (*p* = 0.74)	− 0.16 (*p* = 0.66)	− 0.21 (p = 0.70)	0.21 (p = 0.70)	0.20 (*p* = 0.67)	0.34 (*p* = 0.35)	− 0.55 (*p* = 0.09)
Zn_ptot_					0.44 (*p* = 0.20)	− 0.11 (*p* = 0.75)	− 0.30 (*p* = 0.39)	0.29 (*p* = 0.41)	0.36 (*p* = 0.34)	− 0.11 (*p* = 0.77)	0.13 (*p* = 0.71)

The co-occurrence of considerable amounts of Cd, Cu, Pb, and Zn is typical for areas affected by metallurgical industry. During high temperature, processing of polymetallic ores Cd, Cu, Pb, and Zn undergo volatilization and react with SO_2_, CO_2_, and HCl in the off-gas producing salts. During cooling of the gas, these salts precipitate on the ash surfaces (Fernandez et al. 1992) and the emission of these airborne particles acts as a major transport pathway for trace metals from smelters to soils (Cancès et al. 2003; Czaplicka and Buzek 2011; Wuana and Okieimen 2011). Farmer and Farmer (2000) estimated that in the vicinity of Ust-Kamenogorsk, the spatial extent of wind-driven transport of industrial dust along the predominating E-SE winds was as long as 50 km.

In the soils studied, a large part of the trace metals, except for Cu, was loosely bound (i.e., partitioned to exchangeable and acid-soluble fractions) to the soil matrix. This means that the elements can be released to groundwaters under usual physical-chemical conditions. The contribution of exchangeable and acid-soluble fractions was 18–36% and 20–35%, respectively, for Cd, 3–10% and 18–27% for Zn, 2–11% and 11–50% for Pb, and 1–5 and 1–18% for Cu (Tables 2, 3, 4, and 5; Fig. 4). The relative contributions from the remaining fractions varied considerably. Reducible fraction was the most abundant form of Zn (3–49%). Reducible Pb was between 6 and 22%; Cd ranged from 9 to 15% and Cu from 1 to 21% (Tables 2, 3, 4, and 5). Oxidizable Cu by far predominated (49–64%) and was followed by Pb (3–49%) and Zn (16–28%) (Tab. 2–5). The lowest contents of oxidizable fraction was revealed by Cd (8–17%). Residual fraction for Cd, Zn and Cu varied in a similar range from 11 to 37%, while for Pb it was between 5 and 78% (Tables 2, 3, 4, and 5 ). In general, while the contributions of different fractions of Cd and Zn were comparable, Pb and Cu were highly unevenly partitioned, i.e., some forms of these metals by far predominated over the others. The partitioning of trace metals in the samples was weakly related to physical and chemical features of soils, i.e., the correlations between % contributions from chemical fractions and soil parameters were low and statistically insignificant (data not shown). In addition to it, spatial variability of the % contributions of fractions was low (Fig. 4). We believe such features mirror the partitioning of trace metals in the industrial dust, which is the most important carrier of trace metals in the soils.

**Fig. 5:**
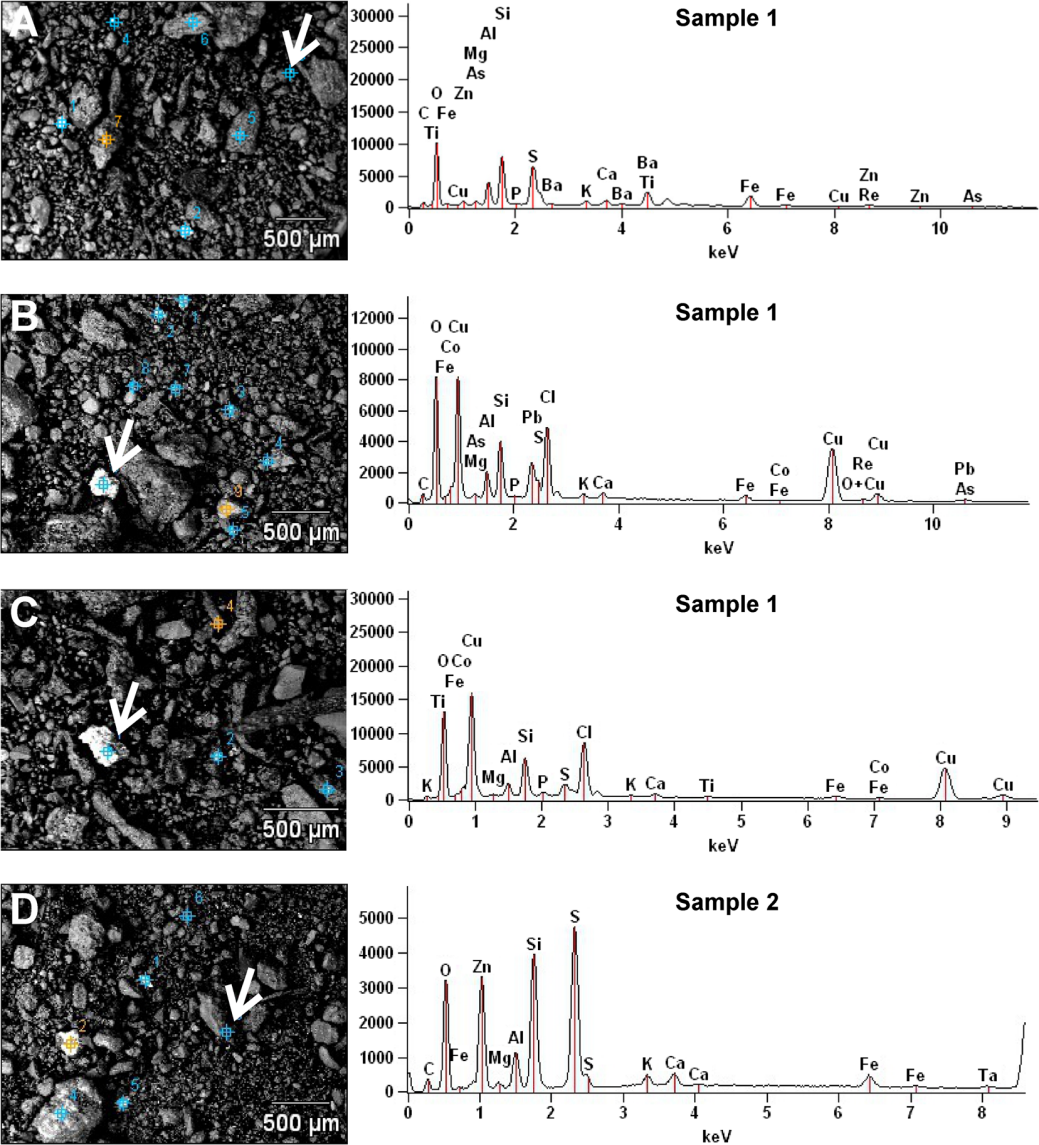
SEM images and EDS spectra of two soil samples analyzed (**a**–**c** sample no. 1; **c** sample no. 2). White arrows indicate grains analyzed with EDS. **a** Polymetallic alloy. **b** Polymetallic alloy and trace metal chlorides. **c** Trace metal chlorides and sulfides. **d** Trace metal sulfides

It has been documented that industrial dust is predominantly composed of reactive and soluble forms of trace metals including chlorides (CdCl_2_, CuCl, PbCl_2_, ZnCl_2_), sulfates (CdSO_4_, PbSO_4_), and carbonates (CdCO_3_, PbCO_3_, ZnCO_3_) (Fernandez et al. 1992; Maskall and Thornton 1998; V. Ettler et al. 2005; Yang et al. 2006; Sammut et al. 2010; Czaplicka and Buzek 2011). These compounds are leachable during the first and second step of sequential extraction (Czaplicka and Buzek 2011) acting as exchangeable and acid-soluble forms of the metals. Reducible, oxidizable, and residual fractions in the dust are usually of minor importance. The former two are mainly composed of metal oxides (CdO, Cu_2_O, PbO, ZnO) and sulfides (CdS, PbS, ZnS), respectively (Sobanska et al. 1999; Sammut et al. 2010; Czaplicka and Buzek 2011) while residual fraction is highly heterogenous and contains polymetallic alloys, ore fragments, and different minerals (Kierczak et al. 2008; Audry et al. 2010). Inspection of the samples using SEM-EDS provide evidences that some of the above phases are likely to occur in the soils studied. Elemental composition of grains indicates the occurrence of polymetallic alloys (Fig. 5a, b), chlorides (Fig. 5b, c), and sulfides (Fig. 5c, d) within silicate matrix. Kucha et al. (1996) showed that within the soil water displaying pH between 6.8 and 8.0, the range covering the pH of the soils studied, many minerals of trace metals, including silicates, are prone to etching. This leads to release of trace metals to soil waters and subsequent precipitation of secondary minerals (predominantly carbonates) and/or scavenging by mineral and organic matter (Ettler et al. 2005; Sipos et al. 2008).

In the weathering zone, the trace metals display selective affinities to different compounds which explain their speciation in soils. For example, Cd forms easily soluble chlorides and substitutes for Ca^2+^ in carbonates (Sammut et al. 2010; Barać et al. 2016) and thus preferentially occurs in exchangeable and acid-soluble fractions. Cu tends to form stable complexes with organic matter (Sipos et al. 2008; Wuana and Okieimen 2011; Montenegro et al. 2015; Radomskaya et al. 2016) and is usually partitioned to oxidizable fraction. In turn, Pb and Zn display well pronounced affinities to clays, carbonates, Fe/Mn oxides, and organic matter which explain equal contributions from different fractions of these metals in soils. Yang et al. (2006) showed that 100% of mobile Pb and 70% of Zn can be fixed by Fe/Mn oxides. Cancès et al. (2003) reported up to 60% Pb and Zn bound to humic substances. Maskall and Thornton (1998), Ettler et al. (2005), and Barać et al. (2016) showed common occurrence of Pb and Zn carbonates in contaminated soils. The residual fraction of these metals is usually of minor importance. The summed contents of exchangeable and acid-soluble factions provide a proxy to assess the mobility of the elements (Zhou et al. 2017). In the soils of Ust-Kamenogorsk, the highest mobility was displayed by Cd, followed by Pb and Zn, while Cu was the least mobile element.

### Assessment of contamination of soils

**Fig. 6:**
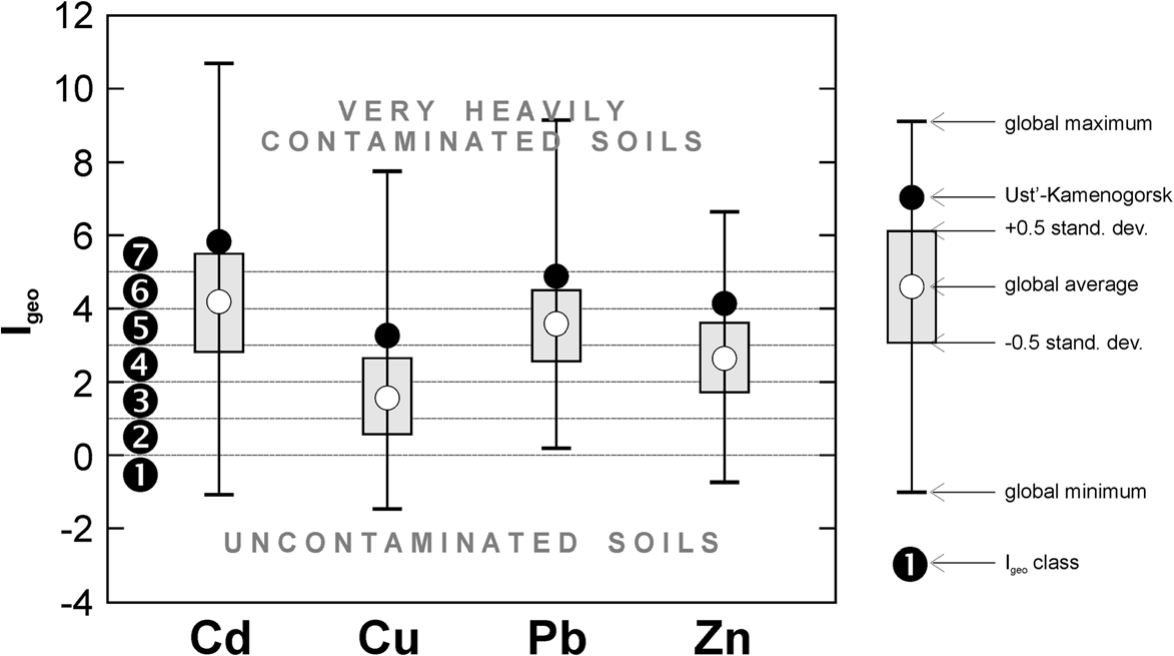
The contamination of soils in Ust-Kamenogorsk in terms of geoaccumulation indices (I_geo_) for the metals analyzed. The data used to produce box-and-whisker diagrams are summarized in Table 7. The numbers from 1 to 7 (left panel) denominate I_geo_ classes (1—uncontaminated soil; 2—uncontaminated to moderately contaminated soil; 3—moderately contaminated soil; 4—moderately to strongly contaminated soil; 5—strongly contaminated soil; 6—strongly to extremely contaminated soil; 7—extremely contaminated soil)

In terms of pollution load index (PLI) of 33.4, it appears that the soils in Ust-Kamenogorsk display heavy polymetallic pollution. The values of geoaccumulation index (I_geo_) show that the contents of Cd, Cu, Pb, and Zn surpass geochemical background values for each element (Tables 2, 3, 4, and 5). The highest I_geo_ of 3.7–6.5, indicative of extreme degree of contamination, is obtained for Cd. For Pb and Zn, the I_geo_ are lower (2.8–5.7 and 2.6–4.6, respectively) albeit the contamination is still strong to extreme. The lowest I_geo_ is for Cu (1.5–4.7), and these values indicate moderate to strong contamination. The PLI in the soils from Ust-Kamenogorsk are among the highest values obtained in industrial and urban soils worldwide (Table 7). The I_geo_ for the elements studied are well above the global average values, specifically for Cu and Zn (Fig. 6). Higher I_geo_ values are displayed in large mining (e.g., Canchaque, Lubumbashi; see Table 7) and smelting centers (e.g., Palmerton, Calcuta, Sambo, Charterhouse; see Table 7). According to Ajmone-Marsan and Biasioli (2010) exceedingly high contents of trace metals occur in the soils of central parts of large cities but the great majority of urban sites have less contaminated soils compared to Ust-Kamenogorsk (Table 7). Despite being very high, the I_geo_ in the soils of Ust-Kamenogorsk displays the overall decreasing trend from the vicinity of the metallurgical complex towards the outskirts of the city.

**Table 7:** An overview of pollution load index (PLI) and geoaccumulation index (I_geo_) values in large industrial and urban centers worldwide. The cities are ordered according to descending PLI. I_geo_ was calculated on the basis on *C*_*n*_ and local *B*_*n*_ reported in publications from the “Reference” column. In case the local geochemical background was not provided in the source publication, the average shale trace metal values (Taylor and McLenan 1995) were used as *B*_*n*_

City	PLI	I_geo_				Reference	Source of pollution
		Cd	Cu	Pb	Zn		
Palmerton (USA)	129	6.9	6.3	5.9	6.6	Ketterer et al. (2001)	Zn smelter
Calcuta (India)*	76	9.7	0.6	8.1	4.3	Chatterjee and Banerjee (1999)	Pb smelter
Canchaque (Peru)*	50	10.6	5.1	3.0	1.6	Bech et al. (1997)	Cu mine
Sambo (Korea)	42	4.3	2.7	6.2	6.1	Jung and Thornton (1996)	Pb–Zn smelter
Lubumbashi (Congo)	36	5.6	7.7	2.8	2.3	Narendrula et al. (2012)	Cu mine
Charterhouse (UK)	33	2.8	0.4	9.1	5.5	Nahmani et al. (2007)	Ancient Pb mine
**Ust-Kamenogorsk (Kazakhstan)**	**32**	**5.7**	**3.2**	**4.8**	**4.0**	**This study**	**Zn smelter**
Hechi (China)	31	3.5	3.7	5.8	4.6	Yuan et al. (2017)	Pb–Sb smelter
Bukowno (Poland)	27	6.0	0.3	4.6	5.9	Verner et al. (1996)	Pb–Zn smelter
Metaleurop/Umicore (France)*	25	6.7	0.3	5.5	3.7	Douay et al. (2008)	Pb–Zn smelter
Shenyang (China)	22	6.0	2.9	3.9	2.7	Li et al. (2013)	Multibranch industry
Avonmouth (UK)	22	5.0	2.0	4.5	4.1	Nahmani et al. (2007)	Pb–Zn smelter
Flin Flon (Canada)	15	3.4	3.6	2.5	3.9	Henderson et al. (1998)	Cd–Cu–Pb–Zn mining/smelting
Miasteczko Śląskie (Poland)*	15	6.4	− 1.5	4.9	3.4	Diatta et al. (2008)	Zn smelter
Bolaroo (Australia)*	14	5.2	0.4	3.8	3.4	Kachenko and Singh (2006)	Pb–Zn smelter
Arnoldstein (Austria)	14	5.2	0.0	5.2	2.4	Friesl-Hanl et al. (2009)	Historical Pb/Zn smelter area
Chenzhou (China)	13	4.1	0.6	4.0	3.8	Huang et al. 2017)	Pb–Zn smelter
Gebze (Turcja)*	11	4.9	0.7	3.3	2.7	Yaylali-Abanuz (2011)	Multibranch industry
Plovdiv (Bulgaria)	11	3.6	1.7	3.1	2.9	Bacon and Dinev (2005)	Pb–Zn smelter
Veles (Macedonia)*	8	5.7	− 0.4	3.1	1.5	Stafilov et al. (2010)	Pb–Zn smelter
Noyell-Gdt (France)	8	4.0	0.1	3.2	2.5	Sterckeman et al. (2002)	Pb–Zn smelter
Shiraz (Iran)*	8	5.1	0.7	3.3	0.2	Qishlaqi and Moore (2007)	Suburban pollutions
Auby (France)	6	3.7	− 0.1	1.5	3.1	Sterckeman et al. (2002)	Zn smelter
Wolverhampton (UK)	6	1.4	2.0	1.2	2.9	Kelly et al. (1996)	Fe industry
Chicago (USA)	4	–	1.8	3.2	1.8	Cannon and Horton (2009)	Urban pollution
Sudbury (Canada)	4	2.8	3.5	0.7	− 0.8	Cannon and Horton (2009)	Urban pollution
Ibadan (Nigeria)	4	0.7	0.3	3.4	1.0	Olajire et al. (2003)	Battery industry
Pribram (Czech)	4	1.3	− 0.2	3.1	1.0	Rieuwerts et al. (1999)	Pb smelter
Oyo (Nigeria)	3	0.8	1.7	1.7	0.7	Olajire et al. (2003)	Fe smelter
Fuheis (Jordan)	3	3.5	–	1.1	0.0	Banat et al. (2005)	Cement factory
Torino (Italy)	3	–	1.1	2.3	1.0	Biasioli et al. (2006)	Urban pollution
Palermo (Italy)	2	− 1.1	0.7	2.2	− 0.2	Biasioli et al. (2006)	Urban pollution
Quezon (Philippines)	2	–	− 1.1	1.8	1.3	Navarrete et al. (2017)	Urban pollution
Gaborone (Botswana)	1	− 1.1	− 0.1	0.3	0.8	Zhai et al. (2003)	Urban pollution
Islamabad (Pakistan)	1	− 0.8	0.5	0.1	− 0.2	Faiz et al. (2009)	Urban pollution

^a^Calculated using average shale trace metal values as *B*_*n*_

The trace metal contents throughout major part of the city exceeded maximum acceptable limits for residential/recreational/institutional areas of 5 mg kg^−1^ for Cd, 100 mg kg^−1^ for Cu, and 500 mg kg^−1^ for Pb and Zn (Beaulieu 2016) (Tables 2, 3, 4, and 5; Fig. 4). Moreover, in major part of the residential zone of Ust-Kamenogorsk, the contents of Zn exceeded the concentration threshold for industrial sites of 1500 mg Zn kg^−1^ (Beaulieu 2016) (Table 5). Consequently, the soils potentially cause a threat to human’s health.

Cd and Pb are among the most severe pollutants (Hutton 1987), and their toxic effects on humans occur at appreciably low doses. The toxic dose for Pb has not been specified because its harmful health effects on humans often occur due to ingestion of very small concentrations of Pb (www.atsdr.cdc.gov). Pb acts as a neurotoxic element and is invoked to cause mental disorder as well as Alzheimer’s and Parkinson’s diseases (Sanders et al. 2009). The toxic dose for Cd was estimated to 1 × 10^−3^ to 5 × 10^−4^ mg Cd kg^−1^ day^−1^ (www.atsdr.cdc.gov). Cd is very biopersistent and displays relatively long biological half-life of > 10 years (Hutton 1987). Its species have documented carcinogenic, teratogenic, and embryocidal effects as well as inflict hypertension and irreversible kidney problems and osteoporosis (Godt et al. 2006). In addition, Cd is more efficiently accumulated by plants than other trace metals (de Livera et al. 2011) which enables Cd intake by humans via gastrointestinal tract. Zn and Cu are less harmful for humans but can be toxic at doses of 0.3 mg Zn kg^−1^ day^−1^ and 0.01 mg Cu kg^−1^ day^−1^ (www.atsdr.cdc.gov). The overdose of Cu and Zn develop psychiatric and endocrinological symptoms and hypotension and causes liver and kidney damage.

Toxicity of Cd and Pb in the soils of Ust-Kamenogorsk is augmented by the mobility of these elements. As shown by sequential extraction, a major part of Cd and Pb is bound to exchangeable and acid-soluble fractions (Fig. 4), which are easily leachable under normal soil conditions and thus become bioavailable (Howard et al. 2013). Davies (1992), Zhang et al. (2014), and Barać et al. (2016) showed experimentally that the concentration of Cd in plants grown in contaminated soil is directly related to the abundance of exchangeable Cd in the soil, and Pb is absorbed in proportion to its total content. In agreement with the above, Farmer and Farmer (2000) found excessive concentrations of Cd, Pb, and Zn in plant material (hay, pasture grasses) and livestock tissues adjacent to Ust-Kamenogorsk.

## Conclusions

In this study, an attempt has been made to assess the pollution of top soils in Ust-Kamenogorsk due to accumulation of trace metals delivered by intense industrial activity. From our data, it appears that the soils in the city are heavily contaminated with Cd, Cu, Pb, and Zn. A large part of these elements is partitioned between exchangeable and acid-soluble forms which implies enhanced bioavailability of trace metals and creates serious ecotoxicological hazard to local biota. The mobility of trace metals in the soils changed in the order Cd > Pb > Zn > Cu, which indicates that the most toxic elements are only weakly bound in the soil. Our data act as one of the very first reports on trace metals in soils from Kazakhstan and Central Asia.
